# Four Ultrasound and Clinical Pictures of Parathyroid Carcinoma

**DOI:** 10.1155/2012/363690

**Published:** 2012-11-14

**Authors:** Milan Halenka, David Karasek, Zdenek Frysak

**Affiliations:** Division of Endocrinology, Third Department of Internal Medicine, University Hospital and Faculty of Medicine and Dentistry, Palacký University of Olomouc, 775 20 Olomouc, Czech Republic

## Abstract

Parathyroid carcinoma is a rare cause of primary hyperparathyroidism. It may be suspected based on severe clinical signs, significant laboratory findings, and the tumor size. High-resolution ultrasonography with Doppler imaging has become the principal imaging method in the preoperative diagnosis of primary hyperparathyroidism. The ultrasound finding is not specific, but three of the described pictures are different from the typical appearance of benign adenoma of the thyroid gland and were suspicious in the context of clinical findings. According to the ultrasound criteria, one finding was benign and only the histological examination revealed carcinoma.

## 1. Introduction

Parathyroid carcinoma (PCa) is a rare cause of primary hyperparathyroidism (PHPT). It is mostly reported to account for 1% of cases; however, the proportion may be as high as 5% in some countries (e.g., Japan and Italy) [1]. The main causes of PHTP are solitary parathyroid adenoma (PAd) in 80 to 85% and multiple adenomas, or multiglandular hyperplasia in 10 to 15% of the cases [2]. On rare occasions, PCa and PAd were found to be concurrent [3]. Although PCa is mostly sporadic, familial occurrence has also been reported [4].

PCa may be suspected based on the severity of clinical signs of PHPT, size of the parathyroid gland, and laboratory findings. PHPT due to a benign lesion is usually characterized by mild symptoms and course. The clinical signs and laboratory findings in PCa are usually marked [4, 5].

High-resolution ultrasonography (US) with Doppler imaging and technetium-99m sestamibi (^99m^Tc-MIBI) scintigraphy are basic approaches to the preoperative diagnosis of PHPT. The two methods, each having its strengths, complement each other and are both characterized by high specificity and a positive predictive value (PPV). If used simultaneously, the PPV ranges from 85% to 90% [6, 7].

## 2. Ultrasound Examination Methods

Ultrasound examination was performed using the Philips Sonos 5500 system and a 10 MHz linear probe, with simultaneous Doppler mapping. A patient was supine, lying on an adjustable bed, with the head tilted back comfortably. The examined area was the frontal part of the neck, from the carotid bifurcation to the superior thoracic aperture, that is, within the probe reach.

The findings typical for PAd are as follows: a solid, homogeneous, hypoechoic mass, with an average size of 16–19 mm (range 4–63 mm) and hypervascularization confirmed by Doppler imaging. It is usually localized in the superior parathyroid, posterior to the upper third of the thyroid lobe, and in the inferior parathyroid, posterior or inferior to the lower pole of the thyroid lobe. The shape is oval, ellipsoidal, or bean-like [8, 9].

On a US scan, PCa may be distinguished from PAd by its size, usually of more than 3 cm, nonhomogeneous structure, lower echogenicity, and frequent degenerative changes—cystic cavities and calcifications and irregular borders [10].

## 3. Case Report 1

The case is interesting in that malignancy was doubled. A search for the etiology of severe hypercalcemia in a patient with relapsed B-cell non-Hodgkin's lymphoma revealed PCa. The 66-year-old male had laboratory-confirmed hypercalcemia 3.41 mmol/L (normal range: 2.06–2.6 mmol/L). When looking for its cause, PHPT was confirmed, with an intact parathyroid hormone (iPTH) level of 616 pg/mL (normal range: 12–65 pg/mL). The clinical signs of PHPT were dominated by recurrent left-sided nephrolithiasis (Table 1). The neck was negative to palpation.

**Figure 1:**
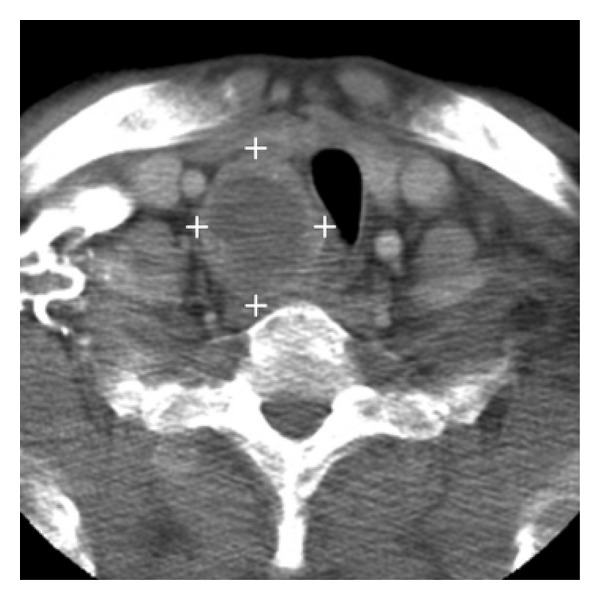
Computed tomography finding of parathyroid carcinoma (transverse)—a cystic tumor in the upper mediastinum, tracheal deviation.

Imaging methods showed a large tumor in the right inferior parathyroid gland. Ultrasound examination revealed the nonenlarged thyroid gland with a normal structure. Under the lower pole of the right lobe, a large cystic tumor was found, sized approximately 5.5 × 4.0 × 4.0 cm, 47 mL in volume, and propagating dorsally and retrosternally. The tumor had a complex echogenic structure, with a hyperechoic solid part and several centrally located anechoic cavities. A CT scan of the neck and upper mediastinum revealed a cystic tumor in the region, sized 5.3 × 4.1 × 3.8 cm, which was well differentiated from the lower pole of the thyroid, propagating into the upper mediastinum up to the aortic arch and causing tracheal deviation (Figure 1). Along the aortic arch, small lymph nodes were observed. Neither US nor CT was conclusive as to whether it was cystic PAd or PCa. However, the finding was rather suspicious. Parathyroid scintigraphy using a complex one-day protocol combining subtraction scintigraphy (^99m^Tc-MIBI and ^99m^TcO_4_^−^) with two-phase technetium-99m sestamibi (^99m^Tc-MIBI) confirmed the presence of hyperplastic parathyroid tissue in the right inferior parathyroid region.

**Figure 2:**
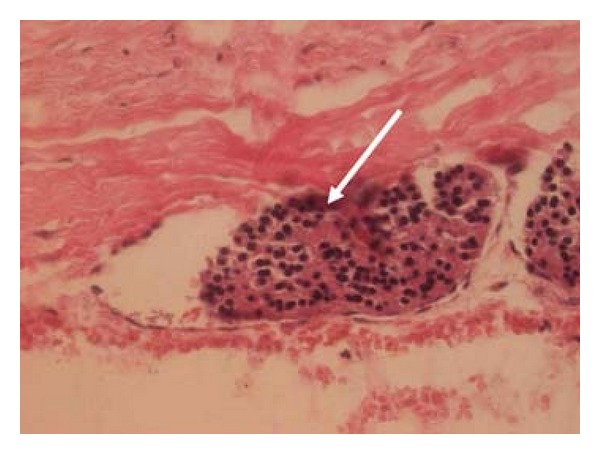
Histological findings of parathyroid carcinoma—vascular invasion (arrow); HE, ×400.

The tumor was completely removed by a surgeon. Histologically, PCa was confirmed (chief cells with focally increased proliferative activity, tumor tissue necrosis, multifocal capsular, and vascular invasion (Figure 2)).

## 4. Case Report 2

A 68-year-old female was referred to the department of internal medicine for a hypercalcemic crisis. Originally, she was admitted to a psychiatric ward for confusion. Laboratory findings revealed hypercalcemia 4.62 mmol/L and confirmed PHPT with an iPTH level of 2479 pg/mL. The clinical signs of PHPT were dominated by those typical for the hypercalcemic syndrome—significant mental changes, dehydration, and impaired renal function (creatinine 147 *μ*mol/L; normal range: 44–104 *μ*mol/L). In her history, no standard manifestations of PHPT were found (Table 1). The neck was negative to palpation.

**Figure 3:**
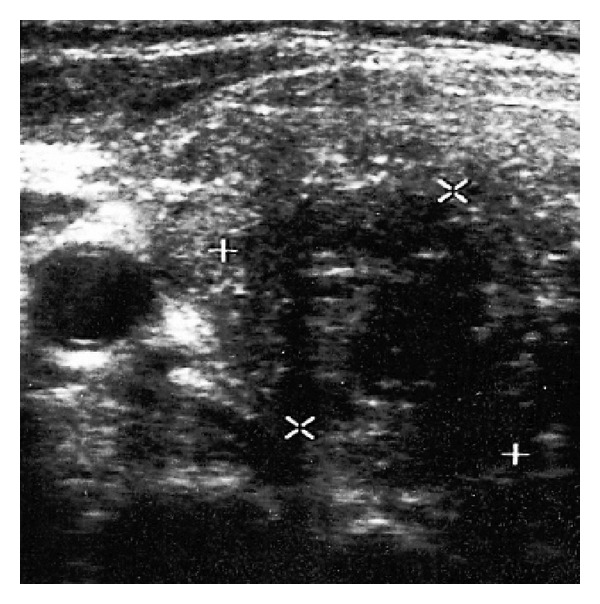
Ultrasound finding of parathyroid carcinoma (transverse)—an intrathyroid, nonhomogeneous, fibrotic mass in the right lobe.

A US scan showed a solid, mostly hypoechoic mass in the lower pole of the right thyroid lobe, with roughened echo structure, increased vascularization, undulate margins, a size of 2.8 × 2.3 × 1.8 cm, and 6 mL in volume (Figure 3). In the context of the PHPT diagnosis, the mass was considered to be an intrathyroidal parathyroid gland. Given its nonhomogeneous structure it was classified as suspicious. Ultrasound-guided fine-needle aspiration biopsy (US-FNAB) was performed. Cytologic smears showed cell clusters with apparent anisonucleosis, atypical nuclei, and only locally present small differentiated cells with clear cytoplasm. The finding was highly suggestive of PCa. Aspirate from the mass showed iPTH above 2500 pg/mL.

Combining subtraction scintigraphy (^99m^Tc-MIBI and ^99m^TcO_4_^−^) with two-phase technetium-99m sestamibi (^99m^Tc-MIBI) revealed only low accumulation of the radiopharmaceutical at the caudal pole of the right thyroid lobe which was considered nonspecific (probably caused by degenerative and neoplastic changes in the parathyroid).

The thyroid was removed completely, including the tumor. Histologically, the finding was described as a clear cell PCa with fibrosis and hyalinization.

## 5. Case Report 3

A 77-year-old female followed for autoimmune thyroiditis and type 2 diabetes mellitus was admitted for an incidental finding of hypercalcemia (s-Ca 3.40 mmol/L). Subsequently, PHPT was confirmed (iPTH 317 pg/mL). Apart from osteoporosis, no other clinical signs were present (Table 1). There were no palpable masses in the neck.

**Figure 4:**
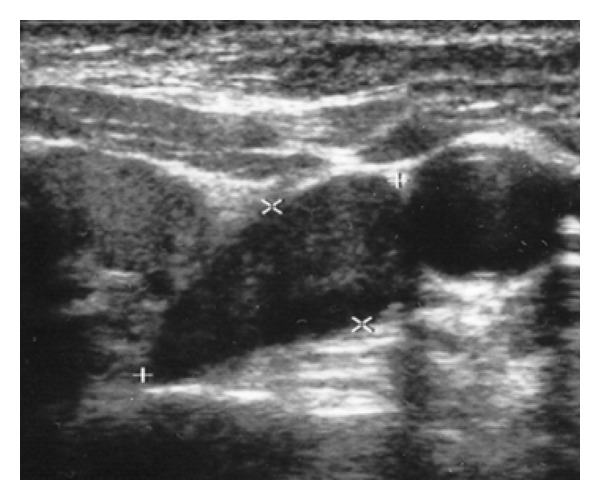
Ultrasound finding of parathyroid carcinoma (transverse)—nonsuspicious “Pad” at the lower pole of the left thyroid lobe.

A US scan revealed thyroid atrophy with autoimmune inflammation (4 mL in volume). Inferior to the lower pole of the right lobe, large hypoechoic PAd was found, with homogeneous structure, increased vascularization, a size of 3.5 × 1.7 × 1.1 cm, and 3 mL in volume. Since the finding was not suspicious according to US criteria, US-FNAB was not performed (Figure 4). Parathyroid scintigraphy was not carried out. Due to severe hypercalcemia, parathyroidectomy of the originally considered PAd was carried out, together with complete removal of the atrophic thyroid. Surprisingly, the histological examination showed PCa from chief cells.

## 6. Case Report 4

A 61-year-old female with a long history of a bipolar disorder was admitted to a psychiatric ward for increasing apathy. Laboratory screening tests showed hypercalcemia (s-Ca 3.47 mmol/L). PHPT was confirmed, with an iPTH level of 2,070 pg/mL. The clinical signs of PHPT in the patient's history included renal colic recurring for about 5 years and acute pancreatitis (Table 1). There were no palpable masses in the neck.

**Figure 5:**
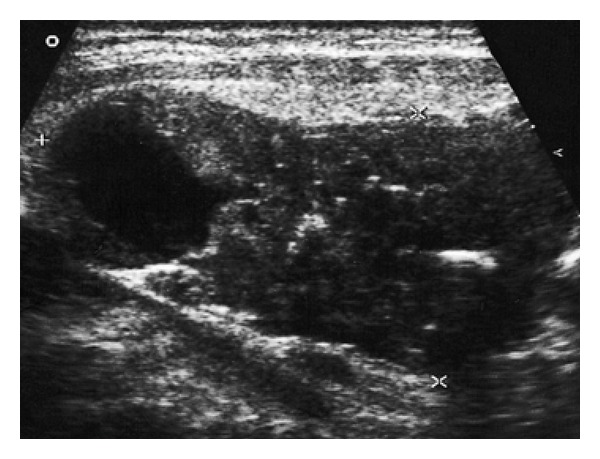
Ultrasound finding of parathyroid carcinoma (longitudinal)—a tumor with cystic degeneration and calcification.

A US scan showed no thyroid abnormalities but in the left inferior parathyroid gland, there was a large tumor with cystic changes and calcifications, sized 4.4 × 2.5 × 1.8 cm and with a volume of 10 mL (Figure 5). US-FNAB was performed. Cytological examination suggested PCa and iPTH levels of more than 2,500 pg/mL were determined in the aspirate. Parathyroid scintigraphy was not carried out. The US scan revealed bilateral nephrolithiasis.

Parathyroidectomy of the tumor was performed. Histological examination showed PCa with bleeding and necroses.

## 7. Discussion

It is difficult to diagnose PCa preoperatively as the clinical signs and laboratory findings are nonspecific. High-resolution US with Doppler imaging and ^99m^Tc-MIBI scintigraphy are the basic methods used for a preoperative diagnosis of PHPT. The two methods, each having its strengths, complement each other.

In the current era of minimally invasive surgery, high-resolution US with Doppler imaging has become the principal imaging method. In recently studied large groups of patients (mostly 100–300 individuals) operated for PHPT, the US sensitivity was 67–96% and its PPV was 82–98%; the ^99m^Tc-MIBI sensitivity was 67–92% and its PPV was 86–91% [6–9, 11]. US examination is maximally utilized in the cases of solitary entopic PAd whereas it is much less utilizable in ectopic PAd. In these large groups of patients operated for PHPT, PCa was found only in two cases reported by Whitson and Broadie [7]. Ectopic PAd (upper mediastinum, retroesophageal, tracheoesophageal groove) is less frequently (20–33% of cases) demonstrated by US. This is when ^99m^Tc-MIBI scintigraphy proves beneficial [6, 9]. All authors of the cited studies were consistent in that both US and MIBI are less sensitive in the case of multigland parathyroid hyperplasia. Moreover, US is less utilizable in multigland hyperplasia, with some 20% of positive findings in the cited groups [6]. In abnormal findings (localization or echo structure), US-guided FNAB may also be performed. The iPTH assay from aspirate is also highly beneficial, providing differentiation from other sites (thyroid nodule and lymph node) and thus contributing to a correct diagnosis [12].

Generally, the advantages of US are low costs, repeatability and no radiation load. Although there is a risk of subjective bias, it may be of a high value if performed by an experienced sonographer. It must be realized that US is not meant to diagnose PHPT (the diagnosis is made clinically and from laboratory results) but to localize the source of PTH overproduction. Therefore, the examining sonographer (usually a radiologist or possibly an endocrinologist skilled at US) needs to have precise clinical information available. US enables a simultaneous morphological assessment of the thyroid gland and an investigation of a concomitant thyroid disease and the surrounding area, in particular enlarged lymph nodes. US may be used for precise measurements of the PAd size (volume) and the description of its structure. Abnormal findings may be suggestive of parathyroid tumors or cysts.

Parathyroid carcinomas are generally larger than adenomas, occasionally with a palpable mass in the neck. But as they affect mainly the inferior parathyroid gland and propagate into the mediastinum, they may be impalpable. According to the National Cancer Data Base 2000 review, in a group of 286 cases of PC reported over an 11-year period, the average size of tumors was 3.3 cm; regional nodes were enlarged in 36.7% of cases but only in 15% of those metastases were found [13]. In another group of 17 patients, carcinomas were sized 2–7 cm [14].

The conclusions of large studies of patients operated for PHPT are consistent in that the basic signs of suspected PCa include markedly increased serum levels of calcium and iPTH, severe clinical manifestation of PHPT, and often palpable tumor mass in the neck [15, 16]. Somewhat surprising and controversial are the results published by Chang et al. who reported 8 cases of PCa in a group of 168 patients over a 12-year observation. Their calcium and iPTH levels were slightly lower when compared with those in benign lesions. Moreover, the patients had no clinical signs of hypercalcemia and 5 of them had tumors smaller than 3 cm [17].

Three cases reported in this paper demonstrate the major advantage of US, that is, its ability to assess the enlarged parathyroid gland morphologically and suggest neoplasia in the context of clinical findings. As far as the tumor size as a sign of malignancy is concerned, both a large tumor (Case reports 1 and 4) and small PCa of less than 3 cm were recorded (Case report 2). Of great benefit was US-FNAB to determine iPTH levels in the aspirate. ^99m^Tc-MIBI scintigraphy was successful in confirming the presence of hypertrophic parathyroid tissue in a large tumor despite its cystic degeneration. However, the finding was not equally clear in a small fibrotic PCa, probably due to the fact that fibrosis limited adequate accumulation of the radiopharmaceutical. ^99m^Tc-MIBI scintigraphy certainly does not allow to suspect potential neoplasia. Other imaging methods such as computed tomography or magnetic resonance imaging need to be used for preoperative diagnosis in case of large tumors involving the upper mediastinum (Case report 1).

However, one case (Case report 3), probably that of early PCa, showed that, in the US examination, even the established “benign” criteria are not 100 percent reliable for PAd. Moreover, the laboratory findings were not suggestive of malignancy and only the histological examination confirmed malignant involvement of the parathyroid gland. It must be realized that, in some cases, PCa may only be diagnosed retrospectively, following local recurrence or metastatic involvement, and, that for a pathologist, it may be difficult to distinguish benign degenerated adenoma from carcinoma histologically [1, 4].

## 8. Conclusion

Clinical suspicion of PCa results from severe clinical symptoms and marked progressively increasing levels of serum calcium and iPTH. The US finding is nonspecific, but, more importantly, the appearance of the tumor is not typical for benign PAd. In most cases, a large tumor in the parathyroid region is revealed (although the size is not specific either), occasionally propagating to the upper mediastinum and often with degenerative changes. In atypical or borderline cases, US-FNAB enables the iPTH level assay in the aspirate and cytologic examination.

## Figures and Tables

**Table 1:** Laboratory and clinical findings.

	Case 1	Case 2	Case 3	Case 4
Age (years)	66	68	77	61
Gender	Male	Female	Female	Female
Clinical findings	Nephrolithiasis	Dehydratation Mental change Renal injury	Osteoporosis	Nephrolithiasis Mental change Acute pancreatitis
s-Ca (mmol/L)	3.41	4.62	3.40	3.47
iPTH (pg/mL)	616	2479	317	2070
Tumor size (mL)	47	6	3	10

s-Ca: calcium serum level and iPTH: intact parathormon serum level.

## Abbreviations

PAd: Parathyroid adenoma
PCa: Parathyroid carcinoma
PHPT: Primary hyperparathyroidism.

## References

[1] Shane E (2001). Clinical review 122: parathyroid carcinoma. *Journal of Clinical Endocrinology and Metabolism*.

[2] DeLellis RA, Mazzaglia P, Mangray S (2008). Primary hyperparathyroidism: a current perspective. *Archives of Pathology and Laboratory Medicine*.

[3] Pai SI, Goldstein BJ, Studeman KD, Westra WH, Tufano RP (2006). Concurrent sporadic parathyroid adenoma and carcinoma. *American Journal of Otolaryngology*.

[4] Cheah WK, Rauff A, Lee KO, Tan W (2005). Parathyroid carcinoma: a case series. *Annals of the Academy of Medicine Singapore*.

[5] Wynne AG, Van Heerden J, Carney JA, Fitzpatrick LA (1992). Parathyroid carcinoma: clinical and pathologic features in 43 patients. *Medicine*.

[6] Haber RS, Kim CK, Inabnet WB (2002). Ultrasonography for preoperative localization of enlarged parathyroid glands in primary hyperparathyroidism: comparison with 99mtechnetium sestamibi scintigraphy. *Clinical Endocrinology*.

[7] Whitson BA, Broadie TA (2008). Preoperative ultrasound and nuclear medicine studies improve the accuracy in localization of adenoma in hyperparathyroidism. *Surgery Today*.

[8] Ulanovski D, Feinmesser R, Cohen M, Sulkes J, Dudkiewicz M, Shpitzer T (2002). Preoperative evaluation of patients with parathyroid adenoma: role of high-resolution ultrasonography. *Head and Neck*.

[9] Abboud B, Sleilaty G, Rabaa L (2008). Ultrasonography: highly accuracy technique for preoperative localization of parathyroid adenoma. *Laryngoscope*.

[10] Hara H, Igarashi A, Yano Y (2001). Ultrasonographic features of parathyroid carcinoma. *Endocrine Journal*.

[11] Boudreaux BA, Magnuson JS, Asher SA, Desmond R, Peters GE (2007). The role of ultrasonography in parathyroid surgery. *Archives of Otolaryngology*.

[12] Sacks BA, Pallotta JA, Cole A, Hurwitz J (1994). Diagnosis of parathyroid adenomas: efficacy of measuring parathormone levels in needle aspirates of cervical masses. *American Journal of Roentgenology*.

[13] Stewart AK, Bland KI, McGinnis LS, Morrow M, Eyre HJ (2000). Clinical highlights from the National Cancer Data Base, 2000. *Ca-A Cancer Journal for Clinicians*.

[14] Moran CA, Suster S (2005). Primary parathyroid tumors of the mediastinum: a clinicopathologic and immunohistochemical study of 17 cases. *American Journal of Clinical Pathology*.

[15] Castillo L, Poissonnet G, Haddad A, Guevara N, Santini J, Demard F (2000). Parathyroid carcinoma: diagnosis and treatment. *Revue de Laryngologie Otologie Rhinologie*.

[16] Pelizzo MR, Piotto A, Bergamasco A, Rubello D, Casara D (2001). Parathyroid carcinoma. Therapeutic strategies derived from 20 years of experience. *Minerva Endocrinologica*.

[17] Chang YJ, Mittal V, Remine S (2006). Correlation between clinical and histological findings in parathyroid tumors suspicious for carcinoma. *American Surgeon*.

